# *BAP1* mutation is a frequent somatic event in peritoneal malignant mesothelioma

**DOI:** 10.1186/s12967-015-0485-1

**Published:** 2015-04-16

**Authors:** Hakan Alakus, Shawn E Yost, Brian Woo, Randall French, Grace Y Lin, Kristen Jepsen, Kelly A Frazer, Andrew M Lowy, Olivier Harismendy

**Affiliations:** Department of General, Visceral and Cancer Surgery, University of Cologne, Köln, Germany; Division of Genome Information Sciences, Department of Pediatrics and Rady Children’s Hospital, University of California San Diego, La Jolla, CA USA; Moores UCSD Cancer Center, 3855 Health Science Drive, Maildrop 0820, 92093 La Jolla, USA; Division of Surgical Oncology, Department of Surgery, University of California San Diego, La Jolla, CA USA; Department of Pathology, University of California San Diego, La Jolla, CA USA; Institute for Genomic Medicine, University of California San Diego, La Jolla, CA USA; Division of Biomedical Informatics, Department of Medicine, University of California San Diego, La Jolla, CA USA

**Keywords:** Genomics, Mutations, Tumor Suppressor, Peritoneal, Mesothelioma

## Abstract

**Background:**

Malignant mesothelioma (MM) arises from mesothelial cells that line the pleural, peritoneal and pericardial surfaces. The majority of MMs are pleural and have been associated with asbestos exposure. Previously, pleural MMs have been genetically characterized by the loss of *BAP1* (40-60%) as well as loss of *NF2* (75%) and *CDKN2A* (60%). The rare peritoneal form of MM occurs in ~10% cases. With only ~300 cases diagnosed in the US per year, its link to asbestos exposure is not clear and its mutational landscape unknown.

**Methods:**

We analyzed the somatic mutational landscape of 12 peritoneal MM of epitheloid subtype using copy number analysis (N = 9), whole exome sequencing (N = 7) and targeted sequencing (N = 12).

**Results:**

Peritoneal MM display few copy number alterations, with most samples having less than 10 Mbp total changes, mostly through deletions and no high copy number amplification. Chromosome band 3p21 encoding *BAP1* is the most recurrently deleted region (5/9), while, in contrast to pleural MM, *NF2* and *CDKN2A* are not affected. We further identified 87 non-silent mutations across 7 sequenced tumors, with a median of 8 mutated genes per tumor, resulting in a very low mutation rate (median 1.3 10^−6^). *BAP1* was the only recurrently mutated gene (N = 3/7). In one additional case, loss of the entire chromosome 3 leaves a non-functional copy of *BAP1* carrying a rare nonsense germline variant, thus suggesting a potential genetic predisposition in this patient. Finally, with targeted sequencing of *BAP1* in 3 additional cases, we conclude that *BAP1* is frequently altered through copy number losses (N = 3/12), mutations (N = 3/12) or both (N = 2/12) sometimes at a sub-clonal level.

**Conclusion:**

Our findings suggest a major role for *BAP1* in peritoneal MM susceptibility and oncogenesis and indicate important molecular differences to pleural MM as well as potential strategies for therapy and prevention.

**Electronic supplementary material:**

The online version of this article (doi:10.1186/s12967-015-0485-1) contains supplementary material, which is available to authorized users.

## Background

Malignant mesothelioma (MM) is an aggressive tumor, which arises from mesothelial cells that line the pleural, peritoneal and pericardial surfaces [1]. The age-adjusted incidence of MM in the USA is about 1 per 100,000 [2]. The majority of MM cases are caused by asbestos or erionite exposure with a latency of typically 20–40 years from exposure to diagnosis. MM is highly resistant to conventional cytotoxic therapies and no active molecularly targeted agents have been identified. As the disease is typically identified late in its course, most patients usually die within 2 years of diagnosis with a total of ~3,000 deaths in the US per year [3,4]. One of the largest studies to date screened 53 primary pleural MM for genome-wide copy-number-aberrations (CNAs) and performed targeted sequencing of selected potential driver genes from the recurrent CNAs [5]. The majority of samples showed recurrent losses of 9p21, 22q and 3p21. *BRCA1* associated protein-1 (*BAP1)* located at the epicenter of 3p21.1 was inactivated by somatic alterations in 42% of all tumors. This study also confirmed findings from previous reports, showing that *CDKN2A* (9p21) and *NF2* (22q) are inactivated in ~60% and 75% of pleural MM respectively [6-10]. Finally other genes have been shown to be mutated in a smaller fraction of MM including *LATS2* (10-30%) which is associated with activation of the YAP pathway [11].

While most studies have focused on pleural MM, about one in ten cases of MM arises from the peritoneum, which makes it an extremely rare condition (incidence ~1 per million in the U.S) and to our knowledge, the largest molecular study to date included only 6 cases [12]. In contrast to pleural MM, about 50% of peritoneal MMs do not have a clear history of asbestos exposure [13] and it is still unknown whether MMs from different sites of origin (pleural, peritoneal or pericardial) share genomic alterations or undergo similar oncogenic transformations [14-16]. Moreover, while whole exome sequencing has been applied to multiple common and rare tumors, it has never been used to determine the genome-wide mutational landscape of MM. Thus we sought to study the mutations present in peritoneal MM using a combination of whole exome sequencing (mutations), copy number arrays (CNA) or targeted sequencing. Examining 12 unique cases of epithelioid peritoneal mesothelioma, we identified the most recurrent somatic events present in this malignancy and compare the findings with what is known about pleural mesothelioma.

## Methods

### Samples and histology

The acquisition and use of peritoneal MM samples was approved by the Institutional Review Board of the University of California, San Diego. Before enrolling in the study, patients gave informed consent. Blood samples for germline DNA extraction were collected before and tumor samples were collected during surgical tumor resection. The resected tumor samples were fixed in 10% formalin, embedded in paraffin and H&E-stained for evaluation by a surgical pathologist. Smaller parts of each tumor were put into 2x2x2 cm wells (Tissue Tek, Miles Scientific), covered with OCT and flash frozen. These samples were used for isolation of tumor DNA after cryosectioning, H&E-staining and evaluation for tumor cell content. Histologic examination of cases of mesothelioma may show many morphologic patterns and variable degrees of cytomorphologic atypia. The main histologic subtypes include epithelioid and sarcomatoid. Our study is limited to the epithelioid subtype. Genomic DNA was extracted from tumor samples with ≥ 70% tumor cell content using the AllPrep® DNA/RNA/miRNA kit (Qiagen®) and germline DNA was extracted from 100 μl buffy coats with the DNeasy Blood and Tissue kit (Qiagen®) according to the manufacturer’s instructions. DNA concentration was determined by fluorometry (Qubit®, Life Technologies).

### Exome capture and library preparation

The sequencing libraries were prepared and captured using SureSelect Human All Exon V4 kit (Agilent Technologies) following the manufacturer’s instructions. Briefly, 500 ng DNA was fragmented by Adaptive Focused Acoustics (E220 Focused Ultrasonicator, Covaris, Woburn, Massachusetts) to produce an average fragment size of ~175 base pairs. Fragmented DNA was purified using the Agencourt AMPure XP beads (Beckman Coulter, Fullerton, CA, USA). The quality of the fragmentation and purification was assessed with the Agilent 2100 Bioanalyzer. The fragment ends were repaired and adaptors were ligated to the fragments. The resulting DNA library was amplified by using manufacturer’s recommended PCR conditions: 2′ at 98°C followed by 6 cycles of (98°C 30”; 65°C 30”; 72°C 1’) finished by 10’ at 72°C. 500 ng of each library was captured by solution hybridization to biotinylated RNA library baits for 48 hrs at 65°C. Bound genomic DNA was purified with streptavidin coated magnetic Dynabeads (Invitrogen, Carlsbad, CA) and further amplified to add barcoding adapters using manufacturer’s recommended PCR conditions: 2′ at 98°C followed by 12 cycles of (98°C 30”; 57°C 30”; 72°C 1′) finished by 10′ at 72°C.

### Exome sequencing and analysis

Sequencing was performed using the Illumina HiSeq 2000 system, generating 100 bp paired-end reads. All raw 100 bp paired-end reads were aligned to the human genome reference sequence (hg19) using BWA v0.5.9-r16 [17] with default parameters for paired-end reads except for seed length set to 35. Aligned reads were realigned using GATK’s [18] IndelRealigner v 1.6-5-g557da77 combining all reads from the same patients and subsequently splitting them. Duplicate reads were removed using Picard Tools v 1.65 MarkDuplicates. Finally the GATK’s TableRecalibration tool was used to recalibrate the reads’ base quality scores. Additional file 1: Table S1 presents the summary statistics of the sequencing. The sequencing data is publically available via the NCBI Short Read Archive (SRA067608). We used VarScan2 v 2.3 [19] to compare the tumor to the normal sample and identify, for each patient, single nucleotide variants (SNVs) and small insertions and deletions (indels) that are: 1) inherited (germline variants); 2) acquired in the tumor (somatic mutations) as well as variants resulting from a loss of heterozygosity (LOH) or of unknown status. The required pileup files for VarScan2 were generated using SAMTools [20] mpileup v 0.1.18 with default parameters except for –q 5, −Q 0, −d 50000, and -B. We used the default parameters for filtering variants except changing the tumor, normal, and combined minimum coverage to 10X each, minimum mutant allele frequency of 0.1, and minimum average quality score to 17. We then applied additional filtering steps. 1) *Low quality indels:* somatic indels with <10X coverage depth or fewer than 3 supporting reads or with more than 5% frequency in the germline are removed. 2) *VarScan default filters:* 2a) Variant within 3 bp of an indel, 2b) clustering SNV: ≥3 SNVs located within 10 bp, 2c) less than 10% allelic frequency. 3) *Low quality somatic variants:* Somatic variants with Varscan Fisher p-value < 0.05 or with >5% alternate allele in the normal DNA (SNVs) or any alternate allele in the normal (indels) are filtered. 4) *VarScan2 high quality filter:* We finally applied VarScan2’s fpfilter script to both germline and somatic variants. This procedure filters variants based on their read position, strand bias, variant reads, variant frequency, distance to 3′, homopolymer, mapping quality difference, read length difference, and mismatch quality sum difference. Variants were queried against dbSNP135 to determine novel or known variants. Next we used snpEff [21] v. 2.0.5 or ANNOVAR [version 2014-07-14] [22] to identify the different the functional and impact on coding genes.

### Illumina exome array and copy number analysis

The tumor DNA from 9 cases was analyzed on the Illumina CoreExome Array. For our analysis, we utilized the Genotyping module (1.9.4) within Illumina’s GenomeStudio (GS) V2011.1. Data was normalized using default parameters and Log R Ratios and B Allele Frequencies were exported for further analysis in R. Because the platform is designed to detect additional rare variants that may interfere with CNA analysis, we used only the probes found on Illumina’s HumanCore BeadChip, which is dedicated to Copy Number Analysis, and excluded the rare variants designed from the human exome. We removed probes with missing values for any of the samples. To perform segmentation analysis, we used the *copynumber* package from Bioconductor using default parameters. For analysis purposes, we assigned a segment with a LogR ratio greater than 0.25 as being an amplified region and a LogR ratio less than −0.25 as being a deleted region. Due to the normalization procedure, chromosome 3 aneuploidy in sample AA2463T was only identified by manual review and added to the segmentation results. A cytoband was called deleted (respectively amplified) when more than 75% of its length belonged to a deleted (resp. amplified) segment.

### BAP1 targeted sequencing and PCR amplification

We used Illumina TruSeq custom amplicons panel. We used DesignStudio (Illumina, Inc San Diego CA) to design custom primers to amplify all exons in *BAP1*. Following the manufacturer’s recommendation we used the DNA from 12 fresh frozen tumor specimens and matched blood DNA from 2 patients to amplify the targets using 14 multiplexing primers. After purification and quantification, we combined the 14 libraries in equimolar amounts and sequenced them using Illumina MiSeq sequencer for 2x150 bp reads. The sequencing reads were then aligned and mutation called using the Illumina BaseSpace cloud with TruSeq DNA amplicon application or using Mutascope locally [23]. To increase the detection sensitivity the reads spanning the expected 42 nt deletion in AA2476T tumor were aligned independently using BLAT [24]. To analyze the large deletion in sample AA2476T we performed the PCR amplification using the following primers: BAP142delF: AGCCAGCATGGAGATAAAGG and BAP142delR TGCCTCAAGGAGGAGGTAGA. The results of the analysis of the BAP1 mutation in AA2476T are presented in Additional file 2: Figure S1.

## Results and discussion

**Figure 1:**
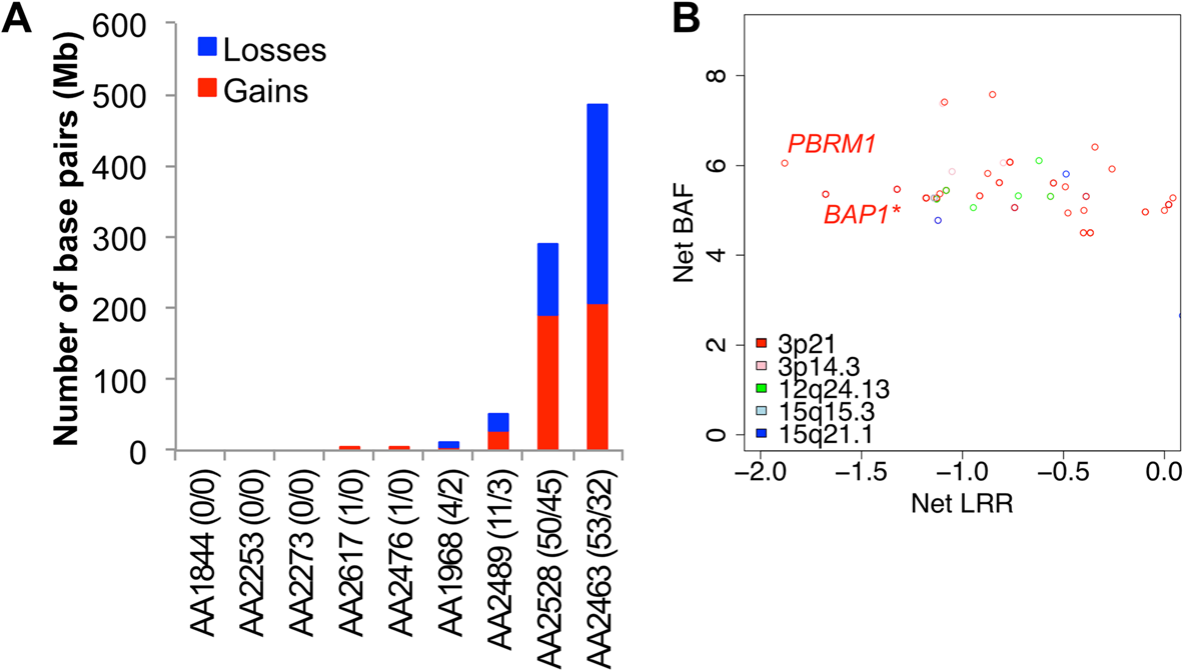
Copy number alterations in 9 peritoneal MM tumors. **(A)** The cumulative number of base pairs affected by losses (blue) or gains (red) in large segments (>1 Mb) is indicated for each tumor. The corresponding number of segments (Lost/Gained) is mentioned next to the sample ID. **(B)** Net Log Ratio (x axis) and B allele frequency (y axis) calculated across all 9 tumors for 369 genes located in the 5 recurrently lost cytobands. 3p21 (red genes) shows the strongest net loss (x axis) and net LoH (y axis). The data points corresponding to *PBRM1* and *BAP1* genes are labeled. (*) the BAP1 data point overlaps with other genes located in the same segment.

To date, most genome-wide analysis of MM have used copy number (or CGH) arrays to identify potentially recurrent chromosomal alterations in the tumors. We analyzed 9 tumors using the Illumina CoreExome arrays, identifying high confidence copy number segments. We observed that 3/9 tumors had no large copy number events, while 3/9 had between 1 and 6 large copy number events, and the remaining 3 had between 14 and 95 segments with a maximum of 290 Mb net copy number changes, including the loss of chromosome 3 in one tumor (Figure 1A – Additional file 1: Table S2). About 14/82 deleted segments also show evidence of a loss of heterozygosity (B Allele Frequency > 0.55), increasing our confidence in these calls. There were no detectable copy number gains recurring in more than 2 samples across the 9 tumors analyzed and none of them were high-level amplifications (max LogR = 0.55). We identified five cytobands where copy number losses or strong loss of heterozygosity are present in 3 or more samples (Additional file 1: Table S3): 12q24.13 (N = 4), 3p21 (N = 3) and 3p14 (N = 3), 15q15.3 (N = 3) and 15q21.1 (N = 3). An analysis of the 369 genes located in these regions reveals that *3p21* harbors the most significantly affected genes, with the lowest net loss (sum of log ratio across all samples) and highest net LOH (Figure 1B). Among those, *BAP1* and *PBRM1*, located 135 kb apart, are known tumor suppressors in pleural MM [5] and renal clear cell carcinoma [25] respectively. At the resolution permitted by our analysis, we did not identify other significant copy number alterations. In particular *NF2* (22q) and *CDKN2A* (9p21), known to be frequently lost in pleural MM do not exhibit any copy number alterations in peritoneal MM (Additional file 2: Figures S3-S4). Overall these results suggest that peritoneal MM exhibits few copy number alterations, mostly losses, and that *BAP1* and its neighboring genes on 3p21 are lost in 5/9 tumors (Additional file 2: Figure S2).

**Figure 2:**
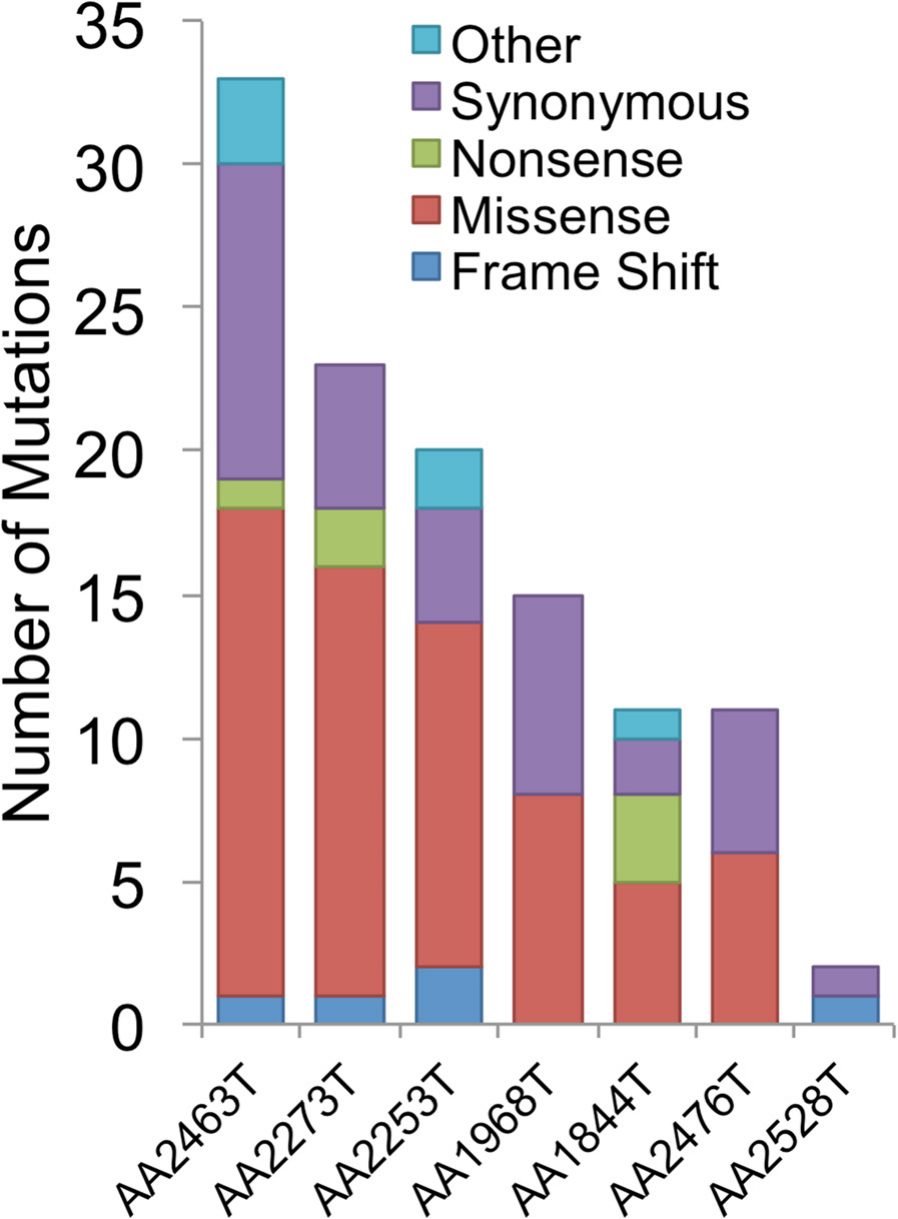
Somatic mutations in 7 peritoneal MM cases. The total number of somatic mutations as well as their predicted effect is represented.

We next performed whole exome sequencing on 7 out of the 9 tumors for which matched normal DNA was available and called somatic mutations in all 7 tumors (Additional file 1: Table S4). We were able to identify between 47 and 133 mutations per tumor, of which 2 to 33 are non-silent (Figure 2). With a median of 1.3 mutations per million base pairs, peritoneal MM has a much lower mutation rate than other adult solid tumors, and comparable to pediatric cancers, leukemias or endocrine cancers [26]. Across all seven cases, 87 somatic mutations were affecting the coding region of 83 genes (Additional file 1: Table S5). *BAP1* was the only recurrently mutated gene, affecting 3 tumors through 2 nonsense mutations and one 42 nt frameshift. The BAP1-K453* mutation has an allelic fraction of 48% in a tumor without *BAP1* loss (AA2273T), indicating that one WT copy remains. Similarly, *BAP1*-Q393* is identified in 12% of the sequencing reads consistent with the genetic heterogeneity of this tumor where a subclonal loss of *3p21* was observed (AA1844T - Additional file 2: Figure S2). Finally BAP1-I71fs was identified in only 14% of the sequencing reads in a tumor with *BAP1* LOH (AA2476T). The unusual length of the deletion (42 nt) may have prevented additional reads from aligning to the reference genome resulting in an underestimation of the allelic fraction. Alternatively, both loss and mutations in this tumor may occur at a sub-clonal level. Overall, our results suggest that *BAP1* is affected by concurrent copy number loss and mutations (2/7), mutation only (1/7) or loss only (3/7).

With the paired sequencing of tumor and normal DNA, we are able to distinguish with confidence germline variants from somatic mutations. In particular, we identified a loss of function variant in *BAP1* (BAP1-Y44*) in the germline DNA of one patient (AA2463T). This patient’s tumor was also characterized by the loss of chromosome 3. The Y44* variant has not been identified in the NHLBI Exome Sequencing Project [27] nor by the Exome Aggregation Consortium [28] and may thus have a minor allele frequency of less than 10^−5^, or be a *de novo* acquired variant in this patient. Inherited loss of function variants in *BAP1* are known to increase susceptibility to melanoma, renal cell carcinomas and malignant mesothelioma [29-31]. A more detailed evaluation of this patient’s family history would however be necessary to establish the true genetic risk associated with this nonsense variant.

To further establish the prevalence of somatic mutations in peritoneal MM, we performed targeted sequencing of *BAP1* exons in 12 tumors, including 3 cases not evaluated by exome or copy number analysis. This analysis allows us to confidently call mutations at high coverage depth. Across all exons and samples, greater than 91% of *BAP1* coding base pairs were covered by 100 reads or more. We were able to confirm in three samples the presence of BAP1-Q393*, K453* and I71fs at an allelic fraction of 20%, 51% and 20% respectively, consistent with the whole exome sequencing findings (Additional file 1: Table S6). We find additional *BAP1* somatic mutations in two samples: at Q684* (50% allelic fraction) and A95fs (39% allelic fraction). In summary, *BAP1* is the most altered gene in peritoneal MM (Table 1), affecting 66% (8/12) of the studied cases through somatic mutations (N = 3), allelic copy number loss (N = 3), or both (N = 2).

**Table 1:** **BAP1 genetic status (germline or somatic) across all 12 cases**

**Sample**	**Assay** ^**1**^	**BAP1**		
		**Germline**	**LOH**	**Somatic (Allelic Fraction)**
AA1844T	A + E + T		subclonal	Q393* (E:12%, T:20%)
AA1968T	A + E + T			
AA2273T	A + E + T			K453* (E:48%, T:51%)
AA2463T	A + E + T	Y44*	chr3	
AA2476T	A + E + T		15 Mb	I71fs (E:14%, T:20%)
AA2528T	A + E + T			
AA2253T	A + E + T		0.34 Mb	
AA2489T	A + T		54 Mb	
AA2617T	A + T	NA		A95fs (39%)
AA2830T	T	NA	NA	
AA2627T	T	NA	NA	Q684* (50%)
AA2819T	T	NA	NA	

1: A: copy number array; E: whole exome sequencing; T: targeted sequencing.

Peritoneal MM is an extremely rare malignancy and, in contrast to pleural MM, has never been analyzed on a genome-wide scale before. Early cytogenetic and loss of heterozygosity (LOH) analyses of pleural MMs described deletions as the most common cytogenetic aberration, suggesting that the inactivation of tumor suppressor genes residing in these deleted chromosomal regions may be responsible for neoplastic transformation [32]. Losses of 3p21 were described as a common alteration (13/23, 57%) in pleural MM twenty years ago [33] and in a recent study, the *BAP1* tumor suppressor gene at 3p21 was frequently affected by loss, mutations or both (22/53; 42%). It has therefore been concluded that *BAP1* loss drives the selection for 3p21 deletions in pleural MM [4]. Furthermore, germline mutations of *BAP1* in two families have also been shown to predispose to pleural MM [29] and heterozygous *BAP1*^−/+^ mice are more susceptible to mesothelioma than their wild type littermate [34] thus strongly implicating *BAP1* as a central player in MM tumorigenesis,.

Here we report that somatic alterations in 3p21 are also present in the majority of peritoneal MM. Five samples revealed deletions at 3p21 resulting in loss of one or two cancer genes (*BAP1* and/or *PBRM1*) per sample. Further investigation by whole exome and targeted sequencing suggests that *BAP1* is the most significant gene in this region as it is recurrently altered by somatic loss of function mutations in 5 tumors. While *BAP1* is a natural candidate driver in 3p21 given its somatic alterations in pleural MM, *PBRM1* is another potential tumor suppressor in the region. Frequent inactivation *PBRM1* by somatic alterations have been described for kidney clear cell carcinoma (92/227, 42%) based on exome sequencing of 7 and targeted sequencing of 257 tumor samples [35]. However in our study *PBRM1* is recurrently lost in 6 peritoneal MM cases but we did not identify any somatic mutations in the 7 samples sequenced. Thus, *PBRM1* is less likely to be the most relevant gene in 3p21, except perhaps in tumor AA1968T where the 3p21 deletion specifically affects *PBRM1* and not *BAP1*.

Beyond *BAP1*, it is important to determine which other genes or pathways may be involved in peritoneal MM. Surprisingly, while somatic alterations of *NF2* (39/53, 74%) and *CDKN2A* (31/53, 58%) are common in pleural MM [5], they are not observed in any of the 12 peritoneal MM cases we evaluated, despite adequate coverage depth (Additional file 1: Table S7). This suggests an important molecular difference between pleural and peritoneal MM. A recent study demonstrated that, in contrast to wild-type animals, *CDKN2A* loss is not required for tumorigenesis in *BAP1*^+/−^ mice, They further demonstrated that *BAP1* mediates Rb1 expression loss via epigenetic down-regulation independent of *CDKN2A* status [34]. This observation may imply differences in the pathogenesis of pleural vs peritoneal MM in which the prevalence of *CDKN2A* loss differs. In our study, only one case of peritoneal MM had a clear loss of function of both *BAP1* alleles, harboring both a chromosome 3 deletion and a germline nonsense variant. In all other cases, one wild type copy of *BAP1* likely remains or the somatic alterations are only present at a sub-clonal level. Thus, while the mouse model may recapitulate a typical tumor suppressor loss pattern, with one inherited variant and one subsequent somatic loss, it appears that alteration or loss of the second allele may not be required in the majority of sporadic peritoneal MMs. Rather, the presence of a wild type allele in most studied cases, suggests that *BAP1* haplo-insufficiency may lead to peritoneal MM. The analysis of more samples would be required to correlate somatic *BAP1* ploidy, somatic status, Rb1 expression and epigenetic landscape with known clinical and environmental features.

*BAP1* is ubiquitously expressed, and involved in multiple processes such as transcriptional regulation, chromatin remodeling or *BRCA1* mediated mismatch repair [36]. It may be feasible to exploit *BAP1* loss of function in MM using synthetic lethal approaches with PARP inhibitors or HDAC inhibitors for example to leverage a potential defect in DNA repair or chromatin remodeling, respectively. This is a hypothesis that remains untested at this time. However a more systematic investigation of *BAP1* partners and targets will likely be required to reveal effective ways to target *BAP1* loss in MM.

## Conclusion

In conclusion, our report suggests that the loss of *BAP1* is a molecular alteration characteristic of peritoneal MM, occurring in the absence of any other strong recognized oncogenic drivers. Inactivation of *BAP1* was previously identified as a common event in pleural MM but had never been described in peritoneal MM before. The lack of alterations in *NF2* and *CDKN2A* supports the idea of distinct genomic features between pleural and peritoneal MM. Further studies with more samples will be required to determine the molecular consequences and potential association with clinical and environmental features.

## Additional files

Additional file 1: Table S1. Sequencing Statistics. **Table S2.** Number and total size of the large (>1 Mbp) copy number aberrations identified in all 9 samples. **Table S3.** List of cytobands with significant losses or gains. **Table S4.** Whole exome sequencing variants and mutations statistics. **Table S5.** List of non-silent somatic mutations. **Table S6.** BAP1 somatic variants identified by targeted sequencing. **Table S7.** Fraction of coding base pairs covered at 20× or more in BAP1, CDKN2A and NF2 genes in whole exome sequencing data. Additional file 2: Figure S1. BAP1 42 nt frameshift deletion in AA2476T. **Figure S2.** BAP1 Copy Number Analysis. **Figure S3.** CDKN2A Copy Number Analysis. **Figure S4.** NF2 Copy Number Analysis.

## Abbreviations

MM: Malignant Mesothelioma
CNA: Copy Number Aberration
