# Supplementary material for: BAP1 mutation is a frequent somatic event in peritoneal malignant mesothelioma
Source: J Transl Med. 2015 Apr 16;13:122. doi: 10.1186/s12967-015-0485-1 (PMC4422481; doi:10.1186/s12967-015-0485-1)
Supplement: Additional file 2: Figure S1. — BAP1 42 nt frameshift deletion in AA2476T. Figure S2. BAP1 Copy Number Analysis. Figure S3. CDKN2A Copy Number Analysis. Figure S4. NF2 Copy Number Analysis. [file 12967_2015_485_MOESM2_ESM.pptx]

## Slide 1
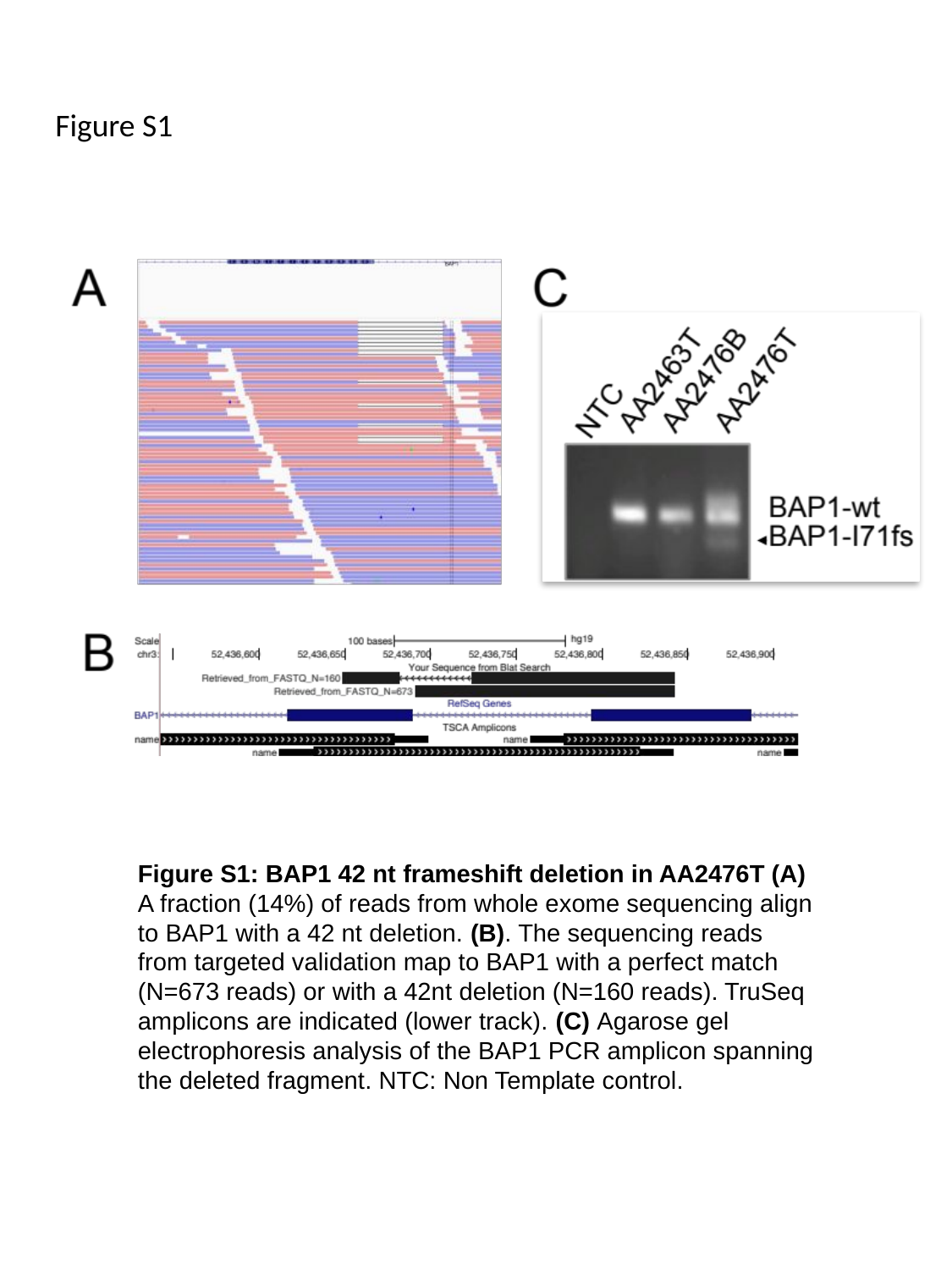

Figure S1
Figure S1: BAP1 42 nt frameshift deletion in AA2476T (A) A fraction (14%) of reads from whole exome sequencing align to BAP1 with a 42 nt deletion. (B). The sequencing reads from targeted validation map to BAP1 with a perfect match (N=673 reads) or with a 42nt deletion (N=160 reads). TruSeq amplicons are indicated (lower track). (C) Agarose gel electrophoresis analysis of the BAP1 PCR amplicon spanning the deleted fragment. NTC: Non Template control.

## Slide 2
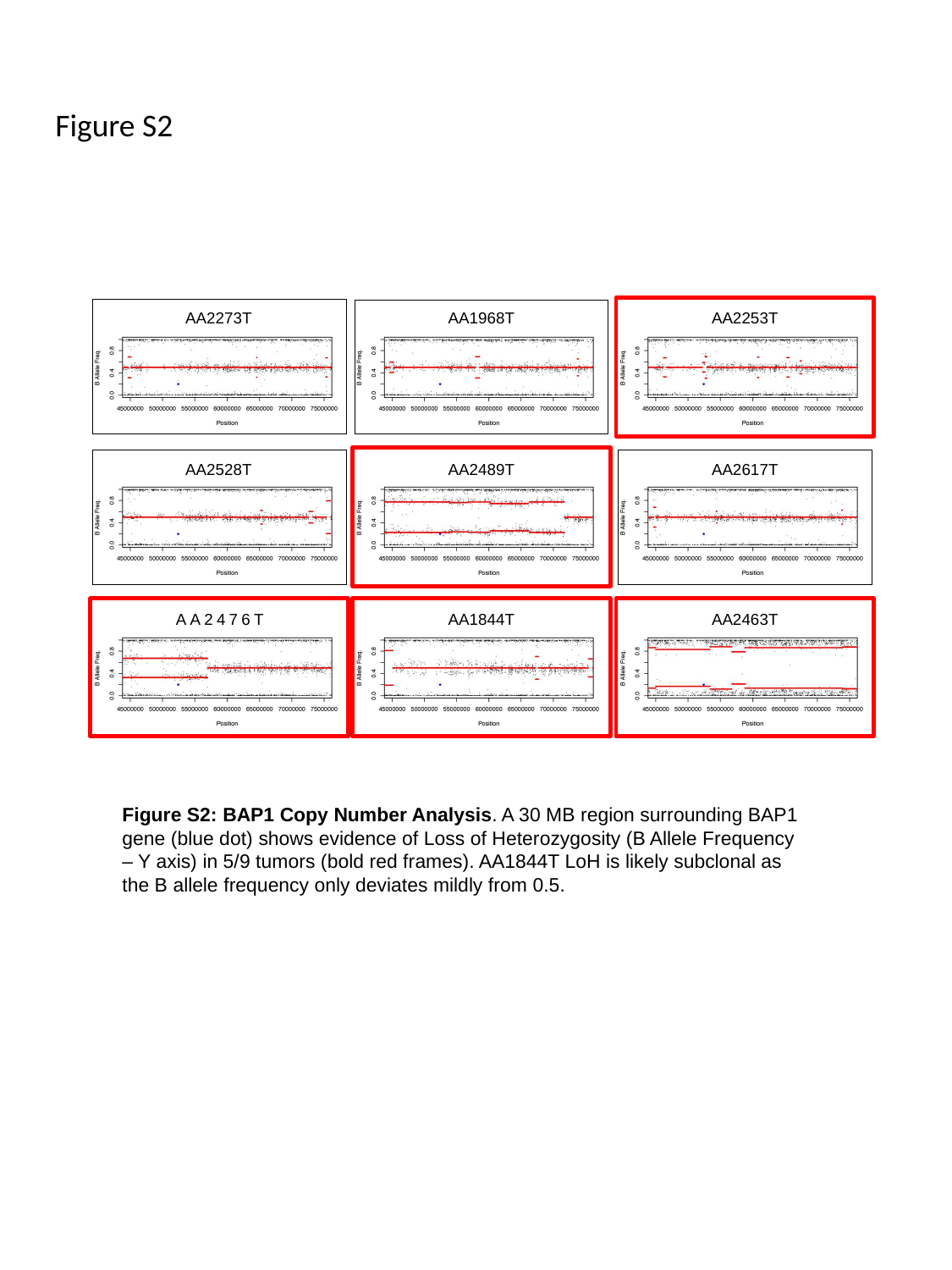

Figure S2
AA2273T
AA1968T
AA2253T
AA2528T
AA2489T
AA2617T
AA2476T
AA1844T
AA2463T
Figure S2: BAP1 Copy Number Analysis. A 30 MB region surrounding BAP1 gene (blue dot) shows evidence of Loss of Heterozygosity (B Allele Frequency – Y axis) in 5/9 tumors (bold red frames). AA1844T LoH is likely subclonal as the B allele frequency only deviates mildly from 0.5.

## Slide 3
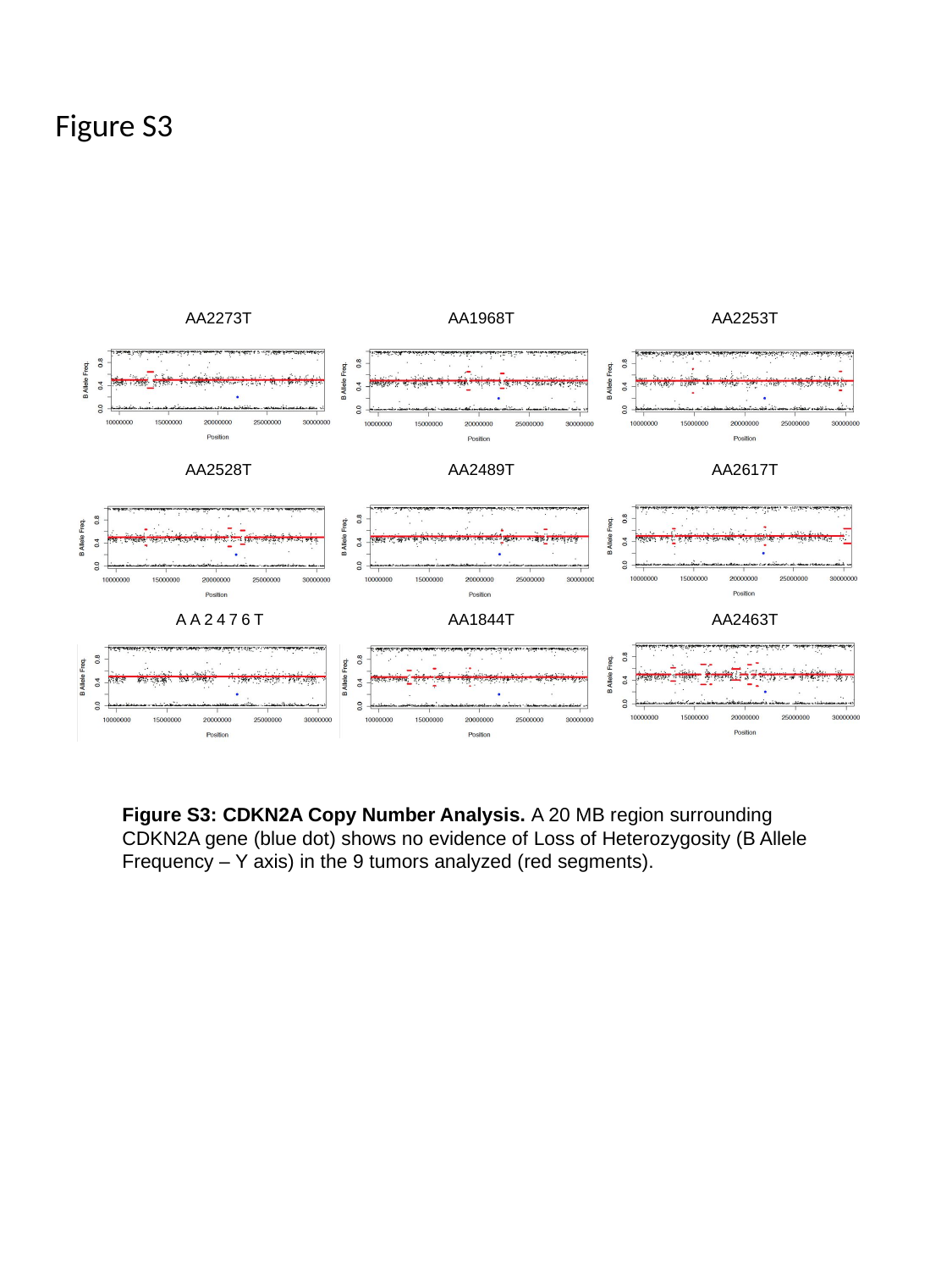

Figure S3
AA2273T
AA1968T
AA2253T
AA2528T
AA2489T
AA2617T
AA2476T
AA1844T
AA2463T
Figure S3: CDKN2A Copy Number Analysis. A 20 MB region surrounding CDKN2A gene (blue dot) shows no evidence of Loss of Heterozygosity (B Allele Frequency – Y axis) in the 9 tumors analyzed (red segments).

## Slide 4
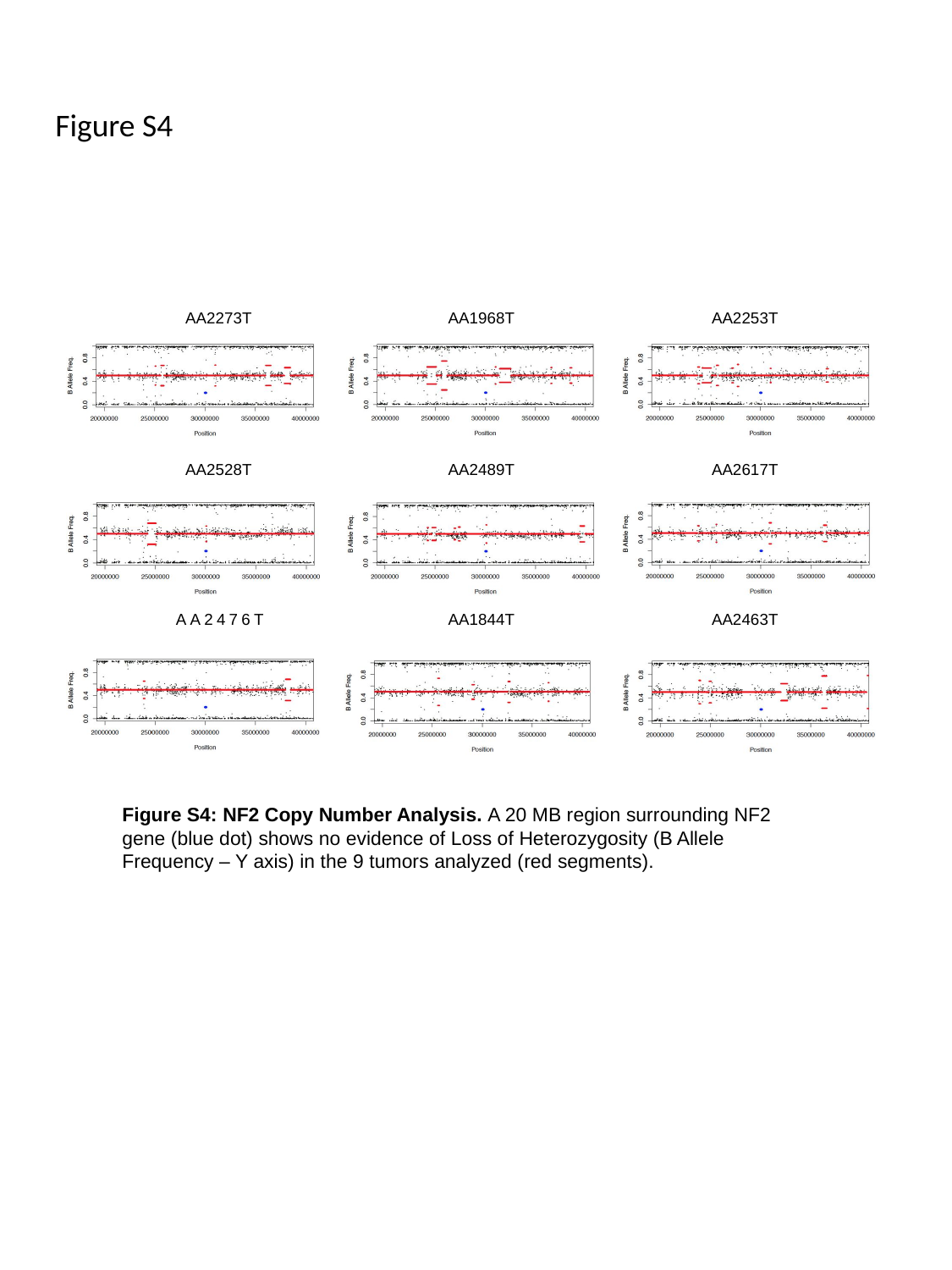

Figure S4
AA2273T
AA1968T
AA2253T
AA2528T
AA2489T
AA2617T
AA2476T
AA1844T
AA2463T
Figure S4: NF2 Copy Number Analysis. A 20 MB region surrounding NF2 gene (blue dot) shows no evidence of Loss of Heterozygosity (B Allele Frequency – Y axis) in the 9 tumors analyzed (red segments).
